# Toward personalizing prosthesis prescription: A take‐home study of three microprocessor‐controlled prosthetic knees: A randomized crossover study

**DOI:** 10.1002/pmrj.70028

**Published:** 2025-10-24

**Authors:** Kinsey Herrin, Sujay Kestur, Sixu Zhou, Gwyn O'Sullivan, Teresa Snow, Walter Lee Childers, Aaron Young

**Affiliations:** ^1^ Woodruff School of Mechanical Engineering Georgia Institute of Technology Atlanta Georgia USA; ^2^ Institute for Robotics and Intelligent Machines Georgia Institute of Technology Atlanta Georgia USA; ^3^ School of Biological Sciences Georgia Institute of Technology Atlanta Georgia USA; ^4^ Extremity Trauma and Amputation Center of Excellence (EACE), Military Performance Lab, Center for the Intrepid Brooke Army Medical Center San Antonio Texas USA; ^5^ Center for the Intrepid, Department of Rehabilitation Medicine Brooke Army Medical Center San Antonio Texas USA

## Abstract

**Background:**

Previous studies on microprocessor‐controlled prosthetic knees (MPKs) often investigate benefits of MPKs as a class of knees rather than clinically relevant differences between specific knees, despite their distinct features.

**Objectives:**

To systematically evaluate and report outcomes associated with three commercially available MPKs following a standardized real‐world use period.

**Design:**

Randomized crossover study.

**Setting:**

Research laboratory and community environment.

**Participants:**

Ten patients with transfemoral amputation.

**Interventions:**

Three MPKs were fitted, trained, and worn for a 1‐week period including C‐Leg 4.0 (Ottobock, Duderstadt, Germany), Rheo Knee‐Model RM7 (Össur, Reykjavik, Iceland), and Power Knee‐PKA01 (Össur, Reykjavik, Iceland).

**Main Outcome Measures:**

Primary outcomes were the 10‐meter walk test (10‐mwt), the 2‐minute walk test (2‐mwt), and the Prosthesis Evaluation Questionnaire (PEQ). Secondary outcomes were stance time asymmetry, physiological cost index, stair and ramp speeds, the narrowing beam walking test, and community ambulation monitoring.

**Results:**

Participants walked 11% faster in Rheo than Power Knee during the 10‐mwt (95% confidence interval [CI]: 0.046–0.184, *p* = .015). In the 2‐mwt, participants walked 12% faster in C‐Leg (95% CI: 0.034–0.241, *p* = .003) and 9% faster in Rheo (95% CI: 0.031, 0.163, *p* = .027) than in Power Knee. On the PEQ, participants reported greater satisfaction with C‐Leg compared to Power Knee (*p* = .006). Ramp ascent speed was 8% faster in Rheo than Power Knee (95% CI: 0.026–0.130, *p* = .024). No significant differences were found for other secondary outcomes. Notably, 10 of 12 outcomes showed individuals performing their best by a defined difference on an MPK different from the cohort's best‐performing MPK.

**Conclusions:**

Participants walked faster in C‐Leg and Rheo than Power Knee and reported greater satisfaction with C‐Leg. Consideration of patient needs and characteristics may allow more individualized MPK prescription and thereby improve rehabilitation outcomes.

**Database Registration:**

NCT06399471.

## INTRODUCTION

Independent community ambulation is challenging for individuals with transfemoral amputation (TFA)^1^ highlighting the need to define prosthetic knee technology benefits for this population. Significant work on microprocessor‐controlled prosthetic knee (MPK) technology showcases benefits of improved gait,^2^ safety,^3^ comfort,^3^ confidence,^3^ balance,^2^ satisfaction,^4^ multitasking,^4^ cost effectiveness,^5^ greater ease in negotiating varying terrains,^3^, ^4^ and reduced falls^4^, ^6^ and energy expenditure.^7^ However, studies associated with MPK technology tend to lump the various knees together as a class^8^ or focus only on a single MPK (the C‐Leg)^2^, ^4^, ^5^, ^6^, ^9^, ^10^ rather than distinguishing benefits of specific knees for individual patients.

A growing body of literature emphasizes the importance of personalized medicine for clinical success^11^, ^12^ and the significance of individual presentation in implementing assistive technology.^13^, ^14^ During prosthesis prescription, patient needs, rehabilitation goals, and environmental factors should be considered, with patients actively involved in device selection.^13^, ^14^ There is a dearth of current evidence comparing the benefits of specific makes and models of MPKs causing clinicians to select based on subjective experiences, intuition,^15^, ^16^ or reimbursement factors. Previous biomechanical comparisons between passive MPKs including C‐Leg and Rheo^17^, ^18^ as well as earlier versions of C‐Leg and Power Knee^19^ offer limited clinical application due to short acclimation periods (2–4 hours) and lack of real‐world testing. Although MPKs are already recognized as an excellent choice for patients based on the aforementioned benefits, a comprehensive understanding of outcomes associated with different types of MPKs could further improve the personalization of MPK prescription to better meet individual needs.

To address these gaps, this study provides a comprehensive assessment of functional and patient‐reported outcomes measures (PROMs) for three modern MPKs—C‐Leg, Rheo Knee, and Power Knee—after a week of community use. C‐Leg uses hydraulic damping, Rheo uses a magnetorheological clutch,^18^ and Power Knee uses a clutchable series‐elastic actuator.^20^, ^21^ The literature suggests C‐Leg offers inherent stability, Rheo allows rapid resistance changes,^22^ and Power Knee assists with ramp and stair ascent and sit‐to‐stand transitions.^21^ However, there remains a lack of up‐to‐date, comprehensively reported, and clinically relevant outcome data on these knees. Therefore, the objective in this study is to systematically evaluate and report such outcomes following a standardized real‐world use period.

## METHODS

### Study design

This was a crossover randomized controlled trial completed over 2 years (October 2021–October 2023) at Georgia Institute of Technology.

### Ethical and regulatory considerations

**FIGURE 1:**
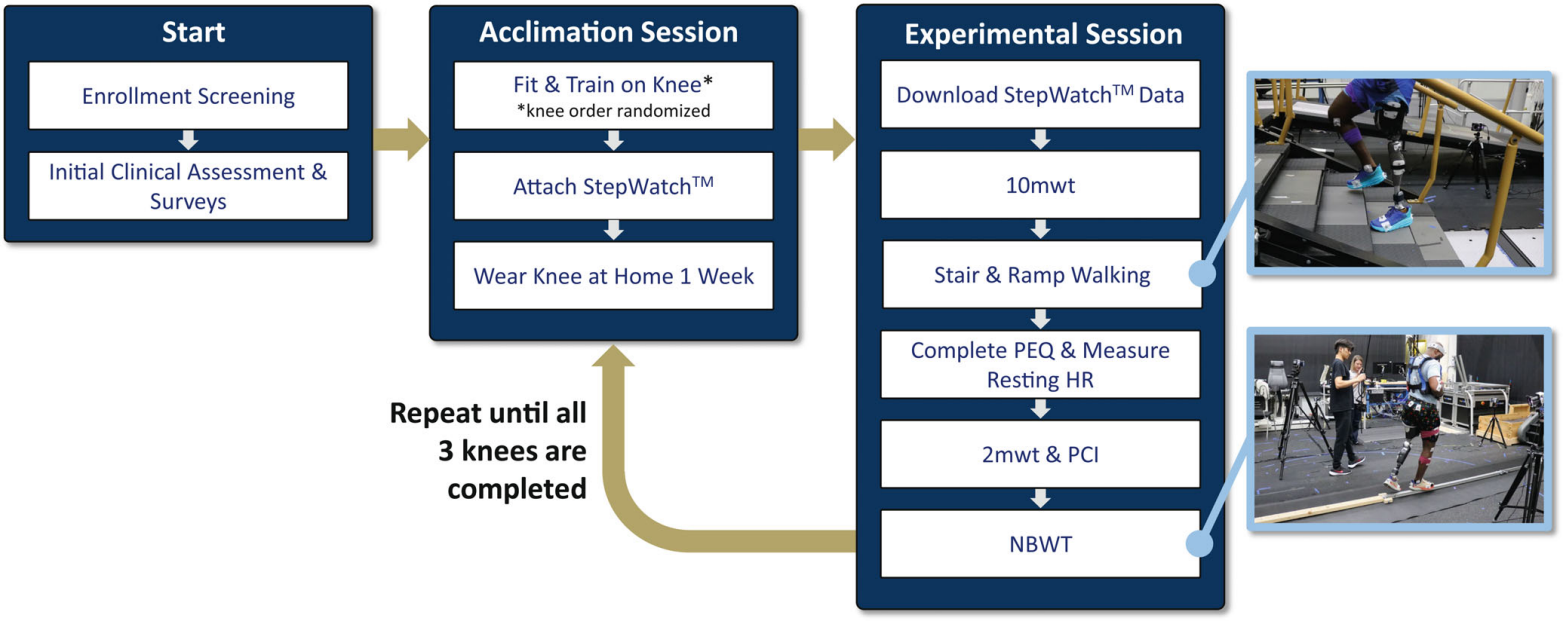
Experimental flow chart dictating experimental protocol. Following enrollment screening, participants underwent an initial clinical assessment and completed surveys on their prosthetic preferences and typical daily/weekly activities. MPK order was randomized in advance and participants were fitted and trained on each knee, which they wore for ~1 week at home prior to returning for an experimental session. Each experimental session for a given knee lasted approximately 1 hour per participant, following which they were also fitted, aligned, and trained with the next knee for testing. This was repeated until experimental sessions for all three knees were completed. 2‐mwt, 2‐minute walk test; HR, heart rate; NBWT, narrowing beam walking test; PCI, physiological cost index; PEQ, Prosthesis Evaluation Questionnaire.

All research was conducted in accordance with the Declaration of Helsinki and the Georgia Institute of Technology Institutional Review Board protocol H21008 (Figure 1), reported according to the Consolidated Standards of Reporting Trials guidelines (Appendix A) and registered at https://clinicaltrials.gov (NCT06399471). Participants provided written informed consent.

### Participants and interventions

Inclusion criteria were age 18–75 years, unilateral TFA, at least 6 months post fitting of a definitive lower extremity prosthesis, daily habitual use of a lower extremity prosthesis, and K3‐K4 ambulators capable of performing all locomotor tasks of interest. Exclusion criteria were significant neuromuscular or musculoskeletal disorder or other comorbidity that would interfere with participation, open wounds on residual limb, known visual impairments that prevent safe operation of a prosthesis, hearing impairments that prevent response to auditory instruction, and current pregnancy due to risk of falling. Three commercially available MPKs, Otto Bock C‐Leg 4.0 (Ottobock, Duderstadt, Germany), Össur Rheo Knee‐Model RM7 (Össur, Reykjavik, Iceland), and Össur Power Knee‐PKA01 (Össur, Reykjavik, Iceland) were used, and participant training was provided for each knee.

### Experimental evaluation procedures

Details on randomization and blinding are found in Appendix B. An initial clinical assessment was conducted including the Amputee Mobility Predictor without Prosthesis (AMPnoPro) outcome^23^ (selected to remove any effects of the clinically prescribed knee on the outcome of the test), basic anthropometric data, and a survey on the participant's daily and weekly activities (Appendix C). The activities survey asked participants to rate the degree of likelihood for performing activities daily and weekly. Activities included standing, walking, or sitting for >30 minutes and sit‐to‐stand transitions, kneeling, lifting, climbing stairs and ramps, running, biking, playing sports, and activity on irregular terrains. Answers were povided on a 1–5 scale where 1 indicated “extremely unlikely” and 5 indicated “extremely likely.”

### Experimental fitting procedures

Participants were fitted, aligned, and trained with their first randomized MPK according to manufacturer standards by the research team including an experienced certified prosthetist. MPK software parameters were adjusted iteratively by the research team while the participant ambulated across level ground, ramps, and stairs prioritizing a natural and comfortable gait with minimal gait deviations. The clinically prescribed suspension, socket, and prosthetic foot were used with the study knee; any clinically prescribed specialty components (eg, rotators and torsion units) were used in the study prosthesis if they could be fit within the build height. If needed, additional prosthetic components were used to achieve proper alignment with each study knee. Only one participant had a clinically prescribed foot that did not fit underneath all study knees, so an Össur LP Proflex was used instead for this participant for all three MPKs. Training included level ground, stairs, and ramps. Participants were permitted to leave with the prosthesis only once they reported feeling safe and comfortable and the prosthetist and study team were satisfied with their performance in the knee. Participants wore the MPK in their community environment for a required minimum weeklong period. A StepWatch activity monitor (Modus, Edmonds, WA) was attached to each participant's prosthetic pylon before leaving the lab.

### Outcome measures

Following the minimum 1‐week wear, participants returned for collection of multiple outcomes with each MPK and the process was repeated for each knee with no washout period between MPKs. Once data collection was complete for all MPKs, we determined both the cohort's optimal performing knee for each outcome as well as each individual's best‐performing knee for each outcome. Primary outcomes were defined as self‐selected walking speed measured through the 10‐meter walk test (10‐mwt) and 2‐minute walk test (2‐mwt), and the Prosthesis Evaluation Questionnaire (PEQ). Other outcomes were secondary outcomes. All outcomes were selected with the intent to capture a comprehensive set of factors that a clinician may consider during MPK prescription. Walking speed is often considered the sixth vital sign and a measure of function, frailty, and independence^24^; ramps and stairs are frequently encountered community barriers^25^; spatiotemporal asymmetry is often a measure of gait quality and can be indicative of future osteoarthritis risk^26^; balance as measured via the narrowing beam walking test (NBWT) is a predictor for falls^27^; physiological cost index as a measure of energy economy may influence a patient's capability in a device^28^; step count and cadence lend understanding for how a patient uses their device in their home environment; and the PEQ importantly captures patient perception of various device features and performance that heavily influence clinical success but are not as clearly captured via functional tests (ie, sounds and aesthetics).

### Level ground walking measures

Speed and stance time (ST) asymmetry index were measured during five trials of the 10‐mwt across an instrumented gait mat (Protokinetics, Havertown, PA). Speed was calculated during the 2‐mwt. In this study, we considered improvements ≥0.08 m/s as a clinically meaningful improvements in speed based on work from Bohannon et al., 2017.^29^

The ST asymmetry index was calculated as follows^26^:

$$\mathrm{ST}\;\text{asymmetry index}=\begin{array}{l} \frac{\text{Prosthetic stance time}-\text{Sound stance time}}{0.5\;\left(\text{Prosthetic stance time}+\text{Sound stance time}\right)}\\ \times 100\% \end{array}$$

A value equal to zero indicates perfect symmetry. Negative values of ST asymmetry index indicate increased time on the sound side, and positive values indicate increased time on the prosthetic side. We defined 7.1% to be a clinically meaningful difference when determining the best knee for an individual based on prior data from a cohort of individuals with TFA.^26^

### Balance measures

Participants were asked to complete the NBWT^30^ in which their furthest distance traversed was recorded for the final three of five total trials. We defined a difference of 3.52 feet to be clinically meaningful^31^ when determining the best knee for an individual.

### Energy expenditure measures

The physiological cost index (PCI) was calculated as follows:

$$\mathrm{PCI}=\frac{\text{Working heart rate}-\text{Resting heart rate}}{\text{Speed}}$$

Participants were required to sit and rest for a minimum of 10 minutes prior to recording resting heart rate. Speed here was calculated from the 2‐mwt, and working heart rate was recorded upon conclusion of the 2‐mwt. We defined a difference of 0.116 beats/m to be clinically meaningful for PCI.^28^

### Community ambulation functional measures

The number of daily steps taken on the prosthetic side and cadence over the ~1 week period were recorded using StepWatch, which is validated for use in individuals with lower limb amputation.^32^ Average daily maximum cadence was recorded for the most intensive continuous 5 minutes/day. When determining an individual's best performing knee, we defined a difference of 1509 steps/day and a cadence of 3.35 steps/minute to be clinically meaningful based on prior data from K3‐K4 ambulators.^33^

Participants completed 10 trials of stair ascent and descent on a six‐step staircase with a 6‐inch step height and 10 trials of ascent and descent on a 5.2° 16‐foot length ramp while speed was recorded. Stair height and ramp grade were selected to match Americans with Disabilities Act recommendations. Participants were encouraged to use the trained stair ascent method but could choose their preferred strategy. Because no published references for stair climbing speeds exist for individuals with TFA, we used the SD of our dataset to set clinically meaningful differences at the individual level: 12.3 steps/minute for ascent and 24.9 steps/minute for descent.

### PROMs

During the rest period for PCI, participants were asked to report the number of falls they experienced and administered a modified PEQ,^34^ which reflected the study use time of 1 week, rather than 4 weeks.

Following completion of all outcome measures, participants were fitted, aligned, and trained as described with the next randomly assigned knee, repeating the procedure in full for all MPKs.

### Data processing and statistical analysis

Primary outcomes of interest (self‐selected speed and PEQ data) were used to determine study sample size. Previously published data were used^35^, ^36^, ^37^ to determine mean differences in individuals with TFA slow and fast walking (1.26 vs. 1.04 m/s) and variability (SD = 0.2 m/s). In addition, Resnik and Borgia (2011)^38^ reported minimal detectable change (MDC) values for PEQ that ranged from 0.8–1.4 with SEs of 0.3–0.633. An MDC of 1.1 and SD of 1.25 were selected for sample size calculation. The power analysis of each variable using G*Power (V.3) for a one‐way, within‐participants design with three levels of measurement indicated a sample size of 10 would achieve 80% power or slightly higher.

A counterbalanced design was used to control order effects and all statistical assumptions were verified to ensure appropriate statistical analyses. Data were analyzed using SPSS (Statistical Package for Social Sciences Version 29.0.0, Chicago, IL). One‐way, within‐participants repeated measures analysis of variance was used to analyze data for both primary (speed, PEQ) and secondary continuous outcomes with sphericity correction (Huynh–Feldt) applied if necessary. Friedman's nonparametric test was used for any continuous variables that violated normality and/or variance assumptions as well as for Likert data. Following significant main effects for MPKs (alpha = .05), post hoc multiple comparison procedures were used to assess differences across MPKs. Uncorrected *p* values from multiple comparisons were adjusted using the Holm–Bonferroni correction method in Excel^39^ to control family‐wise error rate. All significant post hoc comparisons reported in this paper represent corrected *p* values. We used the aforementioned clinically meaningful differences or MDC to determine the best performing knee for each outcome for each individual. When the top‐performing knee differed from the lowest‐performing knee by more than the defined difference, but did not differ from the second‐best knee, both the top and second‐best knees were considered to perform equivalently well for an individual.

## RESULTS

### Participants

Eleven participants provided informed consent, and one participant self‐withdrew immediately following informed consent. All reported data reflect the two females and eight males (age 49.2 [10.8] years, weight 82.37 [17.05] kg, height 1.73 [0.11] m, AMPnoPro 38.1 [3.9]) with unilateral TFA who participated in the full trial. Additional participant demographics, anthropomorphic and AMPnoPro data are shown in Table 1A, and all individual participant data are reported in Table S1.

**TABLE 1:** (**A**) Individual demographics and anthropomorphic data for each participant. (**B** and **C**) Participants’ responses to the daily (**B**) and weekly (**C**) activities surveys in which a score of 1 is indicative of extremely unlikely and a score of 5 is indicative of extremely likely.

A. Demographics and anthropomorphic data																
Participant ID	Height (m)	Weight (kg)	BMI	Gender	Age (y)	Socket type	Suspension	Clinically prescribed knee	Clinically prescribed foot/ankle	Amputated side	Residual limb length	Amputation etiology	Time since amputation (y)	Time in current prosthetic knee	AmpNoPro score	K‐level
S01	1.95	102.00	26.82	M	69	Quad	Suction	Otto Bock C‐Leg	Otto Bock C‐walk	Left	Medium‐long	Iatrogenic	51	3 y	35	3
S02	1.75	74.70	24.39	M	36	Ischial containment	Suction	Plié	Freedom Innovations Renegade AT	Right	Medium	Trauma	5	3 mo	41	4
S03	1.80	80.27	24.78	M	55	Ischial containment	Vacuum	Otto Bock X3	Otto Bock Maverick Xtreme AT	Right	Medium‐short	Trauma	9	5 y	40	4
S04	1.57	53.45	21.68	F	43	Ischial containment	Suction	Plié	Ossur ProFlex	Left	Long	Trauma	25	10 y	42	4
S05	1.79	92.20	28.78	M	44	Ischial containment	Suction	Otto Bock C‐leg 3.0	Fillauer All Pro	Right	Long, knee disarticulation	Blood clot	4	2 y	41	4
S06	1.70	102.70	35.54	M	49	Ischial containment	Suction	Otto Bock C‐leg 4	Ossur LP Variflex with EVO	Right	Long	Diabetes	1	1 y	32	3
S07	1.79	103.35	32.26	M	55	Ischial containment	Suction	Proteor Quattro	Proteor Shockwave	Left	Very long	Trauma	27	2 mo	35	3
S08	1.73	82.65	27.68	M	30	Ischial containment	Suction	Otto Bock C‐leg 4	Otto Bock Trias	Left	Medium	Trauma	3	3 y	42	4
S09	1.55	57.55	23.95	F	59	Ischial containment	Suction	Otto Bock C‐leg 4	College Park Accent	Right	Very short	Osteosarcoma of knee	43	1.5 y	41	4
S10	1.75	74.84	24.44	M	52	Ischial containment	Suction	Otto Bock C‐leg 4	Otto Bock Trias	Right	Medium‐long	Trauma	35	6 y	32	3

B. Daily activities (In a typical day, how likely are you to perform the following activities: 1 indicates “extremely unlikely” and 5 indicates “extremely likely”)													
Participant ID	Stand for an extended period (>30 min)	Walk for an extended period (>30 min)	Sit for an extended period (>30 min)	Go from sitting to standing or standing to sitting	Kneel on the floor/ground	Lift heavy objects	Climb stairs	Walk on ramps	Run	Bike	Play sports	Do water activities	Take your prosthesis on irregular terrains (like sand, grass, rocks, etc.)
S01	No data												
S02	5	5	5	5	2	3	5	2	1	2	2	2	4
S03	5	5	5	5	5	3	3	3	1	4	2	2	3
S04	5	5	3	5	5	4	3	4	5	2	5	2	5
S05	5	3	4	4	4	5	4	4	2	2	3	5	5
S06	5	5	5	5	5	5	5	5	4	3	3	3	5
S07	4	4	5	5	3	3	4	4	1	1	1	2	4
S08	5	5	4	4	3	3	4	4	2	2	3	3	4
S09	3	5	5	5	3	1	5	5	1	3	5	1	5
S10	5	5	3	4	4	5	4	4	2	2	4	3	4

C. Weekly activities (In a typical week, how likely are you to perform the following activities: 1 indicates “extremely unlikely” and 5 indicates “extremely likely”)													
Participant ID	Stand for an extended period (>30 min)	Walk for an extended period (>30 min)	Sit for an extended period (>30 min)	Go from sitting to standing or standing to sitting	Kneel on the floor/ground	Lift heavy objects	Climb stairs	Walk on ramps	Run	Bike	Play sports	Do water activities	Take your prosthesis on irregular terrains (like sand, grass, rocks, etc.)
S01	No data												
S02	5	5	5	5	2	3	5	3	1	1	1	1	5
S03	5	5	5	5	5	3	3	3	1	5	2	2	3
S04	5	5	3	5	4	4	3	4	3	2	5	5	5
S05	4	3	3	4	4	5	4	4	2	2	4	5	5
S06	5	5	5	5	5	5	5	5	4	3	3	3	5
S07	3	3	5	5	3	3	4	4	1	1	1	2	4
S08	5	5	4	4	4	3	4	4	2	2	3	4	4
S09	4	5	5	5	5	2	5	5	1	4	5	1	5
S10	5	5	3	4	4	5	4	4	2	2	4	3	4

### Initial surveys

Data were collected for 9 of the 10 participants on the activities survey due to addition of this survey after enrollment of the first participant. Participants reported the highest likelihoods on average associated with activities of standing and walking for greater than 30 minutes and doing sit‐to‐stand maneuvers on a daily and weekly basis. Taking the prosthesis on irregular terrains and sitting for longer than 30 minutes were also reported as highly likely on a daily and weekly basis. Running and biking were reported as the least likely activities on average. Table 1B,C reports specific responses of individual participants.

### Level ground walking and balance functional outcomes

**FIGURE 2:**
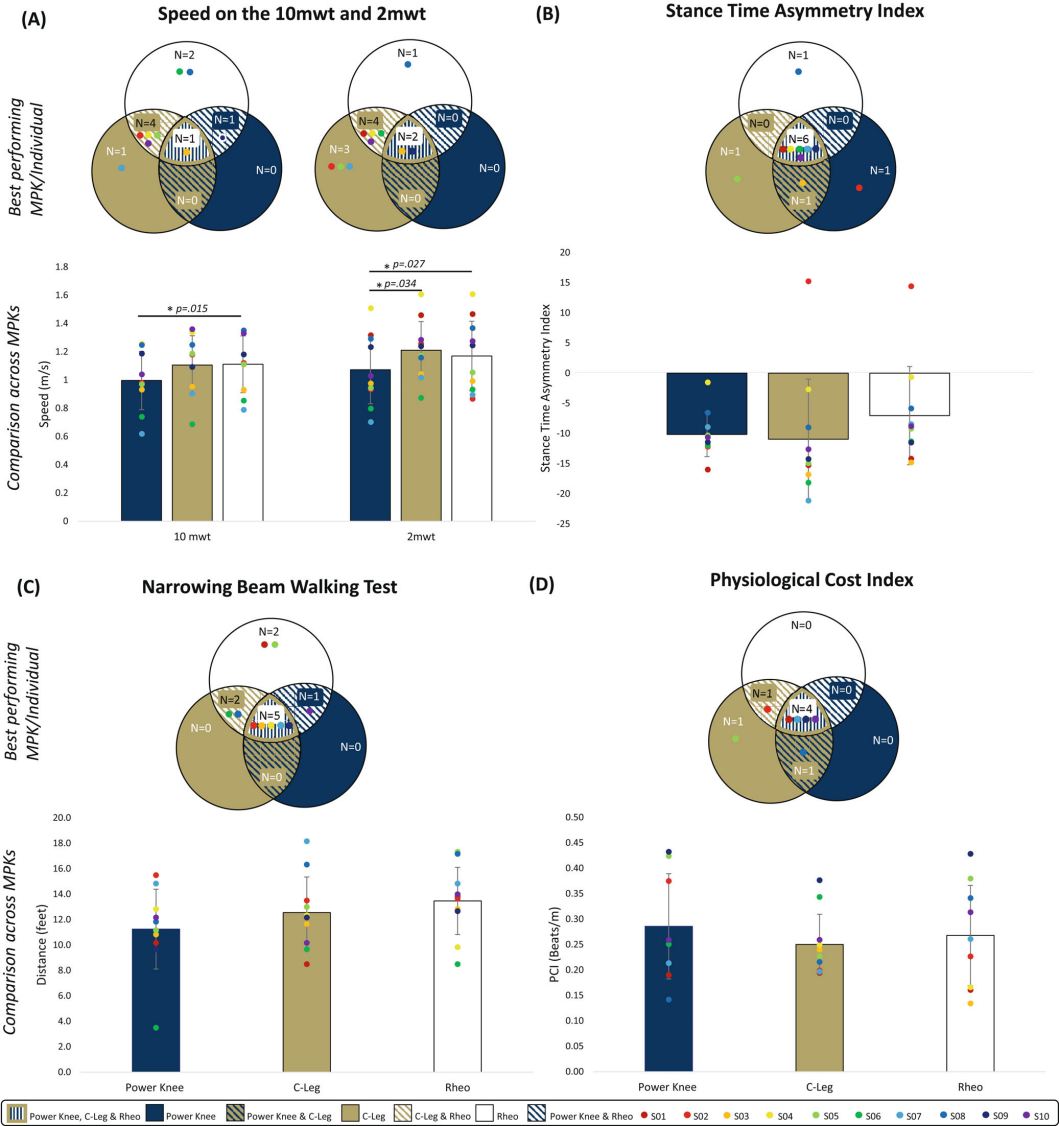
(**A**) Average speed of the 10 mwt and 2 mwt in the Power Knee, C‐Leg, and Rheo. (**B**) Stance time asymmetry index of all three knees during the 10‐mwt. (**C**) Average distance traveled for the final three out of five trials on the narrowing beam walking test. (**D**) Physiological cost index following completion of the 2‐mwt. Venn diagrams represent the best performing knee for N number of participants with those individual participants represented by the colored dot symbol. When all three knees performed equivalently well for a participant, no single best knee was determined and those participants are shown central to the diagram. Bar charts show comparison across the three MPKs. * Indicates significant differences (*p* < .05) between knees. 2‐mwt, 2‐minute walk test; 10‐mwt,10‐meter walk test; MPK, microprocessor‐controlled prosthetic knee; PCI, physiological cost index.

Data were collected for 9 of the 10 participants for the 10‐mwt due to later addition of this outcome. During the 10‐mwt, participants walked on average 10% and 11% faster in C‐Leg and Rheo than in Power Knee (95% confidence interval [CI]: −0.225 to 0.007, *p* = .126 and 95% CI: 0.046–0.184, *p* = .015, respectively) although this difference was not significant when comparing C‐Leg to Power Knee. C‐Leg and Rheo were not significantly different (95% CI: −0.066 to 0.078, *p* = .852). Although most participants walked fastest on either or both C‐Leg or Rheo, one participant (S09) walked equivalently fast on Power Knee and Rheo (Figure 2A). During 2‐mwt, participants walked 12% faster in C‐Leg (95% CI: 0.034–0.241, *p* = .003) and 9% faster in Rheo (95% CI: 0.031–0.163, *p* = .027) than in Power Knee. We observed no significant differences between C‐Leg and Rheo (95% CI: −0.069 to 0.151, *p* = .425). Most participants walked fastest on Rheo or C‐Leg, and two participants (S03 and S09) walked equivalently fast on all three knees (Figure 2A).

The ST asymmetry index was negative in all three knees indicative of increased sound side stance time. On average, Rheo had a 44% and 36% lower ST asymmetry index (improved symmetry) than C‐Leg and Power Knee, respectively, but we observed no statistical differences between knees (*p* = .687) (Figure 2B). Although Rheo was the cohort's optimal performing knee, a high degree of individual performance variability was seen in this measure across knees (Figure 2B).

Participants were able to traverse 18% and 7% further during the NBWT with Rheo than with Power Knee or C‐Leg, respectively (Figure 2C), but we observed no statistical differences (*p* = .082). Most participants performed best on Rheo for this task, but three participants (S06, S08, S10) performed equivalently well to their Rheo performance using one of the other knees.

There were 10 total reported falls throughout 228 days of community use (Table 2). Because not all participants kept each knee for the exact same time due to participant scheduling circumstances, falls are reported as the average falls/day with no statistically significant differences observed (*p* = .943). Four participants (S03, S04, S06, S07) reported no falls on any knee. Three participants (S01, S05, S09) reported the most falls on Power Knee and three participants (S02, S08, S10) reported the most falls on C‐Leg.

**TABLE 2:** Reported falls over the period of time each participant spent on the study knee.

Participant ID	Knee	Falls	Days on knee	Avg falls/d
S01	P	1	7	0.14
S01	C	0	7	0
S01	R	0	7	0
S02	P	0	7	0
S02	C	1	7	0.14
S02	R	0	7	0
S03	P	0	7	0
S03	C	0	7	0
S03	R	0	7	0
S04	P	0	7	0
S04	C	0	10	0
S04	R	0	7	0
S05	P	3	14	0.21
S05	C	0	7	0
S05	R	1	7	0.14
S06	P	0	6	0
S06	C	0	8	0
S06	R	0	8	0
S07	P	0	7	0
S07	C	0	8	0
S07	R	0	5	0
S08	P	0	7	0
S08	C	1	7	0.14
S08	R	0	7	0
S09	P	2	7	0.29
S09	C	0	7	0
S09	R	0	7	0
S10	P	0	14	0
S10	C	1	7	0.14
S10	R	0	8	0

Abbreviations: C, C‐Leg 4.0; P, Power Knee; R, Rheo.

### Energy expenditure outcome

We observed no statistical differences between knees for PCI (*p* = .535). C‐Leg knee had a 13% and 7% lower PCI than Power Knee and Rheo, respectively (Figure 2D). Three data points were removed due to errant experimental heart rate measures. One participant (S05) performed best on C‐Leg, and one participant (S02) performed equivalently on C‐Leg and Rheo. One participant (S08) performed equivalently on C‐Leg and Power Knee, and four participants (S01, S07, S09, S10) performed equivalently across all knees.

### Community ambulation functional outcomes

**FIGURE 3:**
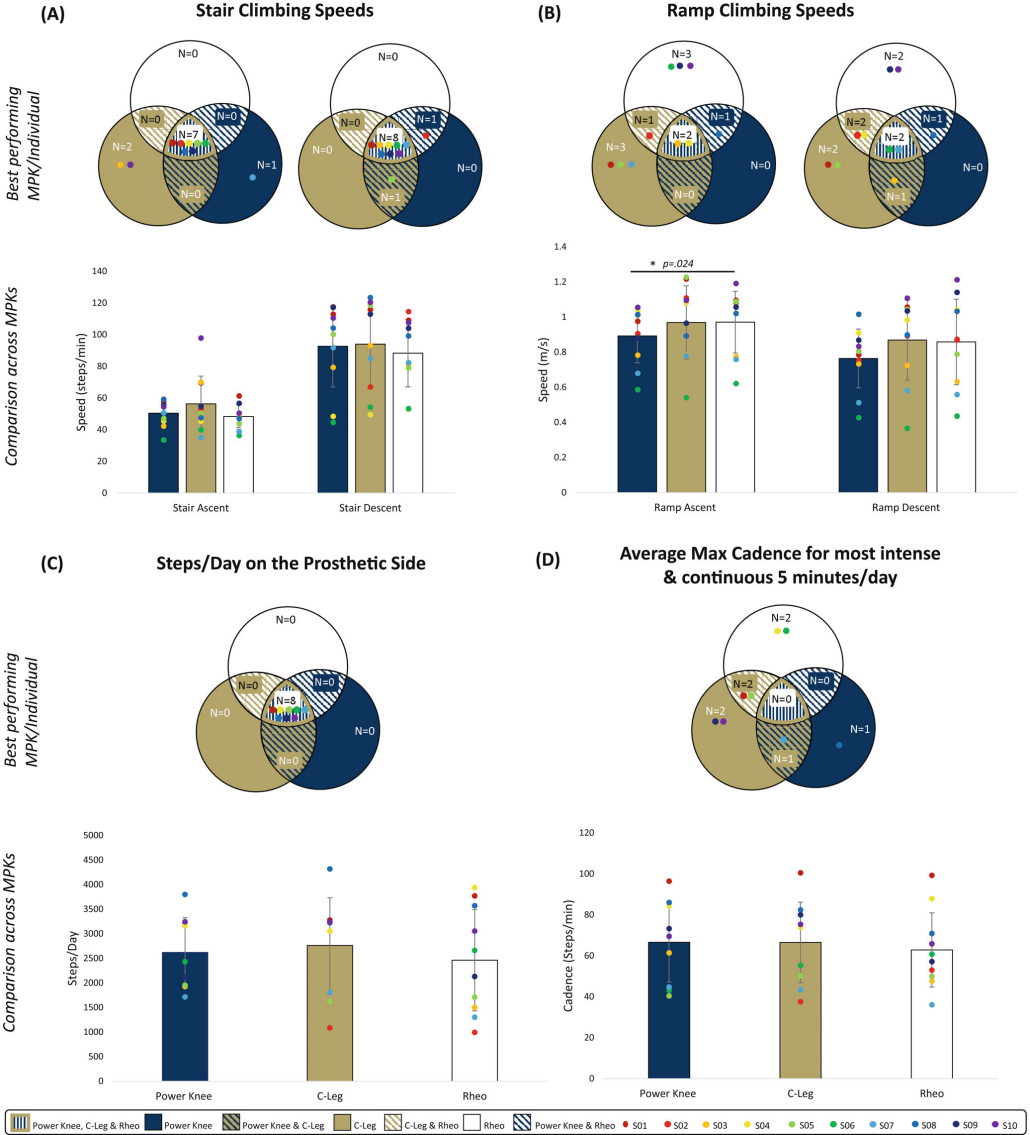
(**A**) Average ascent and descent stair climbing speeds for each knee. (**B**) Average ascent and descent ramp climbing speeds for each knee on a 5‐degree ramp incline. (**C**) Average steps/day as recorded with the StepWatch activity monitor. (**D**) Average maximum cadence for the most intense and continuous 5 minutes per day for each knee. Venn diagrams represent the best performing knee for N number of participants with those individual participants represented by the colored dot symbol. When all three knees performed equivalently well for a participant, no single best knee was determined and those participants are shown central to the diagram. Bar charts show comparison across the three MPKs. * Indicates significant differences (*p* < .05) between knees. MPK, microprocessor‐controlled prosthetic knee.

Stair ascent strategies included step‐to‐step and step‐over‐step patterns with 8 of 10 participants using a step‐over‐step pattern in Power Knee, 8 of 10 participants using a step‐to‐step strategy in C‐Leg, and 6 of 10 participants using a step‐over‐step strategy in Rheo. Participants ascended stairs 11% and 15% faster in C‐Leg than in Power Knee and Rheo, respectively, although these differences were not significant (*p* = .229) (Figure 3A). Although most participants ascended fastest in C‐Leg or Rheo, one participant (S07) ascended fastest in Power Knee. No differences were observed in stair descent speed (*p* = .528) (Figure 3A). Most participants descended equivalently fast on all three knees.

During ramp ascent, participants walked 8% faster in both C‐Leg and Rheo than in Power Knee (*p* = .144 and *p* = .024, respectively) but the difference in C‐Leg and Power Knee was not significant. No differences were observed in speed between Rheo and C‐Leg (*p* = .949). Most participants ascended ramps fastest in C‐Leg or Rheo; one participant (S08) ascended equivalently fast on Power Knee and Rheo (Figure 3B). This same trend held for ramp descent with participants walking 13% and 12% faster in C‐Leg and Rheo, respectively, than in Power Knee with differences approaching statistical significance (*p* = .051). One participant (S03) walked equivalently fast in Power Knee and C‐Leg (Figure 3B).

Complete StepWatch data from all three knees were obtained from eight participants due to experimental issues. Participants took 12% and 5% more steps on the prosthetic side in the C‐Leg than Rheo or Power Knee, respectively; however, differences were not significant (*p* = .367) (Figure 3C). We observed no differences in steps/day between knees for any individual participants. No differences were observed across average max cadence for the 5 most intensive minutes/day for any knee (*p* = .528) with participants performing well on multiple knees (Figure 3D).

### PROMs

**FIGURE 4:**
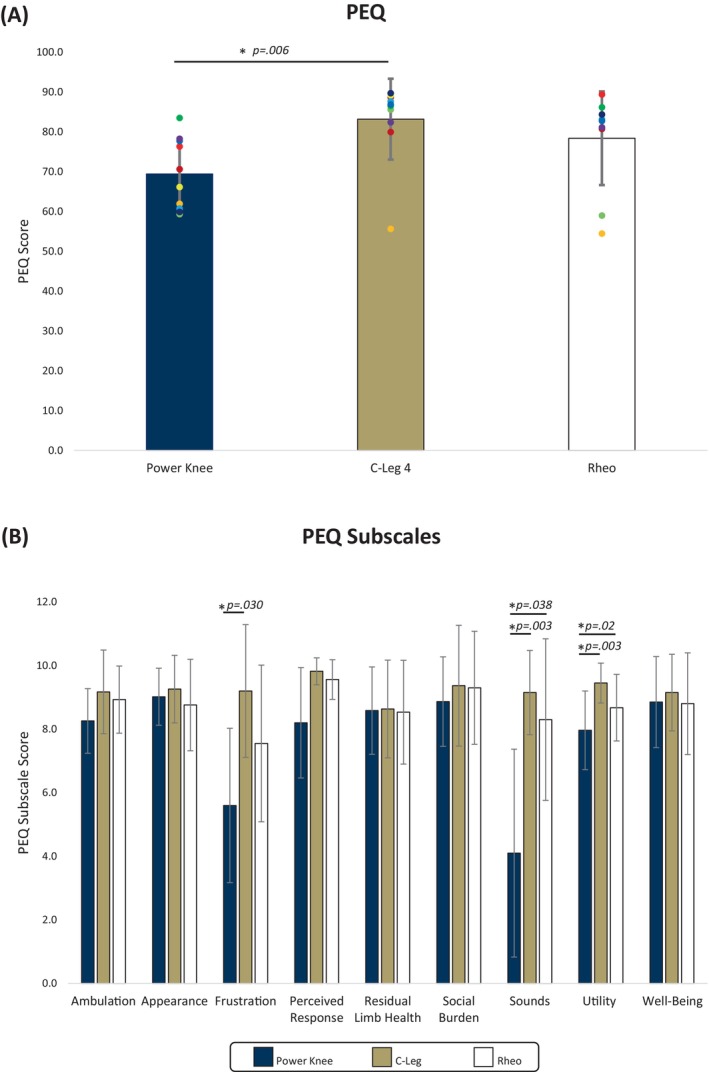
(**A**) Average scores on the Prosthesis Evaluation Questionnaire. (**B**) Average scores on the subscales of the Prosthesis Evaluation Questionnaire. * Indicates significant differences (*p* < .05) between knees. PEQ, Prosthesis Evaluation Questionnaire.

Higher scores indicate improved patient perception on the PEQ. Participants scored C‐Leg and Rheo 18% and 12% higher than Power Knee on the full PEQ (Figure 4A). The median C‐Leg rank (2.7) was significantly higher than the median Power Knee rank (1.3, *p* = .006). There were no differences in Rheo and Power Knee (*p* =.236). On the PEQ subscales (Figure 4B), C‐Leg and Rheo scored significantly more favorably than Power Knee on sounds (*p* = .003 and *p* = .038, respectively). C‐Leg and Rheo scored significantly higher than Power Knee on utility (p = .003 and *p* = .02, respectively). C‐Leg scored significantly more favorably than Power Knee on frustration (*p* = .030). No other subscale was found to be significantly different between knees.

### Safety

No procedure‐related adverse event occurred during this study.

## DISCUSSION

We systematically characterized differences in functional and PROMs between three different commercially available MPKs in 10 experienced users of transfemoral prostheses following a standardized use period in the home and community. We found Rheo was significantly faster than Power Knee in 10‐mwt and ramp ascent, and C‐Leg and Rheo were significantly faster than Power Knee during 2‐mwt. We also found participants reported higher levels of satisfaction in C‐Leg than Power Knee on the PEQ. We found no other statistically significant differences on any other functional outcome measures of speed (ramp descent or stair climbing), energy expenditure (PCI), balance (NBWT and reported falls), or community ambulation functional outcomes (step count and cadence monitoring), which is surprising given the diverse range of outcomes evaluated.

The 2020 Outcomes ASsessment and DISsemination (OASIS) study^40^ compared PROMs retrospectively between four passive MPKs concluding relative equivalent benefits among knees for functional mobility and satisfaction. Our findings somewhat align with this as our measures of speed showed statistical similarities between C‐Leg and Rheo, and we found no other statistically significant differences between outcomes measured. However, the OASIS study measured the PROMs only for a patient in a single passive MPK at an unstandardized point in their use of a device, whereas we measured both functional performance and PROMs across three different MPKs in a single participant at a systematic time point. Further, the cohort in the OASIS study is largely represented by dysvascular amputation etiology whereas our cohort is largely represented by traumatic etiology. Previous studies comparing Power Knee to C‐Leg^19^, ^41^ are now >10 years old and examine only the biomechanics associated with stair climbing and sit‐to‐stand tasks without note of clinical outcomes. One study compared clinical outcomes for prior versions of the Power Knee and Rheo but is also nearly a decade old.^22^ Therefore, this work provides updated comprehensive outcomes for current MPK technology.

Other research studies^17^, ^18^, ^42^ have demonstrated technical and some biomechanical differences among MPK componentry, but these studies have focused comparisons only on passive MPKs without regard to the best performing knee for an individual. However, in 10 of the 12 outcomes we measured, there were individual participants who performed better by a defined clinically meaningful difference on an MPK outside the cohort's optimal MPK. In clinical practice, understanding these differences is important for tailoring prosthesis prescription and progressing a patient's rehabilitation.

A step‐to‐step stair ascent strategy was used more with C‐Leg, whereas participants used step‐over‐step more with Rheo and Power Knee. Although step‐to‐step may be quicker, stair climbing has been linked to osteoarthritis^43^, ^44^ and the step‐to‐step strategy places significant burden on the sound knee.^45^ The step‐over‐step strategy may have been more difficult to learn in the ~1 week of use, leading participants to favor speed over long‐term benefits. This trade‐off choice is critical considering the initial survey data in which this cohort reported high likelihoods of climbing both stairs and ramps in daily and weekly activities. Enhanced training for step‐over‐step stair ascent strategies may reduce the long‐term risk for knee osteoarthritis frequently seen in this population.^44^ Additionally, improved prosthetic design to make stair ascent initiation easier might make this strategy more intuitive, which was a common complaint of our participants.

Our at‐home data monitoring showed minimal differences between knees. Participants maintained consistent cadences and step counts, and no differences were observed in reported falls, possibly reflecting the relative athleticism of our cohort and a continuation of their normal activity and ease at adapting despite changes in their prosthetic knee. This contrasts with previous findings in which participants took fewer steps in a prior version of Power Knee than a prior version of the Rheo,^22^ but that study's cohort was notably ~10 years older.

Participants also reported high likelihoods of activities like standing, walking, sit‐to‐stand maneuvers, and traversing uneven terrains, whereas activities like cycling, running, and playing sports were reported with lower likelihoods. Given the relatively high K‐levels for this cohort, the lack of these higher‐impact athletic activities may be due to lack of secondary prosthetic componentry to support these activities.^46^, ^47^

We found significant differences between C‐Leg and Rheo compared to Power Knee on the PEQ. The subscales revealed reduced levels of frustration, improved utility, and fewer sounds in passive prostheses than Power Knee. These preferences for passive MPKs largely reflect challenges associated with overall control of the device (frustration and utility) and one mechanical design factor (sounds). Concerns with sounds of Power Knee were reported after the week‐long period. This is similar to findings from a previous study comparing prior versions of Power Knee and Rheo.^22^ Additionally, despite receiving training on Power Knee and a week of experience at home, we observed many participants struggle to activate stair mode at the experimental session. This finding underscores the need for devices that automatically recognize varying terrain and properly assist without additional user effort.^48^ Research to blend the controls of passive and active devices together may combine the best of both worlds by using passive‐style control for tasks which require no positive mechanical energy (ie, level ground walking) and active‐style control for tasks that require positive mechanical energy (ie, stair/ramp ascent).^49^, ^50^ These advancements may improve the ability to move seamlessly in community environments.

This study has several limitations including the small sample size and lack of a wash‐out period between knees which was selected due to concerns for participant retention and achieving study timeline. Another confounding factor was that our participant cohort was heavily influenced, experienced and comfortable on passive MPKs. All participants were experienced users of MPKs with 8 of 10 participants being long time (≥1 year) users of Otto Bock MPKs. Participant S07 was a current user (~2 months) of Proteor's Quattro Knee but previously used a C‐Leg for >10 years. Two participants (S02 and S04) had exclusively walked on a nonstudy knee, Plié, a passive MPK. No participant had used or trialed Power Knee. Given the heavy distribution of our participant pool using Otto Bock MPKs in their clinically prescribed prosthesis, there may have been subjective biasing in PROMs toward the study C‐Leg given familiarity with this MPK. Training clearly plays a large role in the success and comfort of a user on a prosthesis^51^ and a single training session and week of acclimation time may be insufficient for embodied use and realization of a prosthesis's benefits. This is especially relevant for major prosthetic changes, such as switching from passive to active technology. Advanced individualized training may accelerate skill adoption and build essential trust and confidence in a new device.

## CONCLUSION

We observed participants walked statistically faster over level ground and ramps while using either or both the C‐Leg or Rheo compared to the Power Knee. In addition, we identified some individuals who performed their best in select outcomes on MPKs that were different from the cohort's optimal‐performing MPK for that outcome. Across the 12 outcomes we studied, 10 outcomes revealed individuals who performed their best beyond the defined clinically meaningful difference on a knee not identified as the cohort's best MPK. These results are in line with a growing body of literature demonstrating individual‐best is not always in line with the average for a given prosthetic component. Future research to elucidate techniques to personalize the MPK prosthesis prescription based on these findings may enhance rehabilitation outcomes for these individuals.

## FUNDING INFORMATION

This work was supported by a grant from the Department of Defense Congressionally Directed Medical Research Program, Orthotics and Prosthetics Outcomes Research Program W81XWH‐21‐1‐0686.

## DISCLOSURE

The authors declare that the research was conducted in the absence of any commercial or financial relationships that could be construed as a potential conflict of interest. Each manufacturer provided knees and knee‐specific training for the study at no charge, however the manufacturers were not involved in funding, study design or the results and interpretation of study data. The results and interpretations presented herein are strictly the findings and opinions of the investigators. The EPIC lab receives funding from Össur for a separate project not related to this study.

This journal‐based CME activity is designated for 1.0 *AMA PRA Category 1 Credit* ^TM^. Effective January 2024, learners are no longer required to correctly answer a multiple‐choice question to receive CME credit. Completion of an evaluation is required, which can be accessed using this link, https://onlinelearning.aapmr.org/. This activity is FREE to AAPM&R members and available to nonmembers for a nominal fee. CME is available for 3 years after publication date. For assistance with claiming CME for this activity, please contact (847) 737–6000. All financial disclosures and CME information related to this article can be found on the Online Learning Portal (https://onlinelearning.aapmr.org/) prior to accessing the activity.

## Supporting information


**Table S1.** All individual participant data for functional performance outcomes where knee types are defined as P=Power Knee, C=C‐Leg 4.0, R = Rheo Knee.

## APPENDIX B Randomization and blinding

The order in which participants tested the three MPKs was randomized prior to enrollment using a random number generator by Researcher 3. Study team blinding was not possible due to need to program all MPKs in manufacturer‐specific software. Participants were also not blinded to MPK type as aesthetics were a part of the assessment within the PEQ.

## APPENDIX C Custom survey administered regarding participant's daily and weekly activities

Daily and weekly activities surveys administered to participants

### C.1. Daily Activities Survey

In a typical DAY, how likely are you to perform the following activities:

Stand for an extended period (>30 minutes)?
 Extremely unlikely.
 Unlikely.
 Neutral.
 Likely.
 Extremely likely.
Walk for an extended period (>30 minutes)?
 Extremely unlikely.
 Unlikely.
 Neutral.
 Likely.
 Extremely likely.
Sit for an extended period (>30 min)?
 Extremely unlikely.
 Unlikely.
 Neutral.
 Likely.
 Extremely likely.
Go from sitting to standing or standing to sitting?
 Extremely unlikely.
 Unlikely.
 Neutral.
 Likely.
 Extremely likely.
Kneel on the floor/ground?
 Extremely unlikely.
 Unlikely.
 Neutral.
 Likely.
 Extremely likely.
Lift heavy objects?
 Extremely unlikely.
 Unlikely.
 Neutral.
 Likely.
 Extremely likely.
Climb stairs?
 Extremely unlikely.
 Unlikely.
 Neutral.
 Likely.
 Extremely likely.
Walk on ramps?
 Extremely unlikely.
 Unlikely.
 Neutral.
 Likely.
 Extremely likely.
Run?
 Extremely unlikely.
 Unlikely.
 Neutral.
 Likely.
 Extremely likely.
Bike?
 Extremely unlikely.
 Unlikely.
 Neutral.
 Likely.
 Extremely likely.
Play sports?
 Extremely unlikely.
 Unlikely.
 Neutral.
 Likely.
 Extremely likely.
Do water activities?
 Extremely unlikely.
 Unlikely.
 Neutral.
 Likely.
 Extremely likely.
Take your prosthesis on irregular terrains (like sand, grass, rocks, etc.)?
 Extremely unlikely.
 Unlikely.
 Neutral.
 Likely.
 Extremely likely.

### C.2. Weekly Activities Survey

In a typical WEEK, how likely are you to perform the following activities:

Stand for an extended period (>30 min)?
 Extremely unlikely.
 Unlikely.
 Neutral.
 Likely.
 Extremely likely.
Walk for an extended period (>30 min)?
 Extremely unlikely.
 Unlikely.
 Neutral.
 Likely.
 Extremely likely.
Sit for an extended period (>30 min)?
 Extremely unlikely.
 Unlikely.
 Neutral.
 Likely.
 Extremely likely.
Go from sitting to standing or standing to sitting?
 Extremely unlikely.
 Unlikely.
 Neutral.
 Likely.
 Extremely likely.
Kneel on the floor/ground?
 Extremely unlikely.
 Unlikely.
 Neutral.
 Likely.
 Extremely likely.
Lift heavy objects?
 Extremely unlikely.
 Unlikely.
 Neutral.
 Likely.
 Extremely likely.
Climb stairs?
 Extremely unlikely.
 Unlikely.
 Neutral.
 Likely.
 Extremely likely.
Walk on ramps?
 Extremely unlikely.
 Unlikely.
 Neutral.
 Likely.
 Extremely likely.
Run?
 Extremely unlikely.
 Unlikely.
 Neutral.
 Likely.
 Extremely likely.
Bike?
 Extremely unlikely.
 Unlikely.
 Neutral.
 Likely.
 Extremely likely.
Play sports?
 Extremely unlikely.
 Unlikely.
 Neutral.
 Likely.
 Extremely likely.
Do water activities?
 Extremely unlikely.
 Unlikely.
 Neutral.
 Likely.
 Extremely likely.
Take your prosthesis on irregular terrains (like sand, grass, rocks, etc.)?
 Extremely unlikely.
 Unlikely.
 Neutral.
 Likely.
 Extremely likely.

Abbreviations: MPK, microprocessor‐controlled prosthetic knee; PEQ, Prosthesis Evaluation Questionnaire.
